# Comparison of ELISA and IFAT for *Leishmania infantum* by European and Middle Eastern diagnostic laboratories

**DOI:** 10.1186/s13071-024-06631-9

**Published:** 2024-12-29

**Authors:** Kurayi G. Mahachi, Marie Ozanne, Patrick Bourdeau, Juliana Sarquis, Eric Kontowicz, Laia Solano-Gallego, Luis Cardoso, Gaetano Oliva, Gad Baneth, Maria Grazia Pennisi, Angela M. Toepp, Guadalupe Miró, Margaret Carrel, Christine A. Petersen

**Affiliations:** 1 College of Public Health, University of Iowa, Iowa City, IA USA; 2 Department of Mathematics and Statistics, Mount Holyoke College, South Hadley, MA USA; 3 Écolecole Nationale Vétérinaire, Agroalimentaire et de l’Alimentation, Nantes-Atlantique (ONIRIS), Nantes, France; 4 LeishVet Association, Veterinary Faculty, Universidad Complutense de Madrid, Madrid, Spain; 5 Animal Health Department, Veterinary Faculty, Universidad Complutense de Madrid, Madrid, Spain; 6 Departament de Medicina I Cirurgia Animals, Facultat de Veterinària, Universitat Autònoma de Barcelona, Bellaterra, Spain; 7 Department of Veterinary Sciences, and Animal and Veterinary Research Centre (CECAV), University of Trás-os-Montes e Alto Douro, Vila Real, Portugal; 8 Department of Veterinary Medicine and Animal Production, University of Naples “Federico II”, Naples, Italy; 9 Koret School of Veterinary Medicine, The Hebrew University of Jerusalem, Rehovot, Israel; 10 Department of Veterinary Sciences, University of Messina, Messina, Italy; 11 Sentara Healthcare, Eastern Virginia Medical School, Norfolk, VA USA

**Keywords:** Dogs, Enzyme-linked immunosorbent assay, Epidemiology, Immunofluorescence antibody test, Latent Class Analysis, *Leishmania*, Leishmaniosis, One Health, Serology

## Abstract

**Background:**

Visceral leishmaniosis (VL) is the most severe form of human leishmaniosis, with an estimated 95% case fatality if left untreated. Dogs act as peridomestic reservoir hosts for the protozoan parasite *Leishmania infantum*, a causative agent for human leishmaniosis, endemic throughout the Mediterranean basin. To assure consistent and accurate surveillance of canine infection and prevent transmission to people, consistent diagnosis of canine *L. infantum* infection across this region is essential for protecting both human and animal health. Our goal was to compare the accuracy, sensitivity and specificity of enzyme-linked immunosorbent assays (ELISA) and immunofluorescence antibody tests (IFAT), performed at seven academic veterinary diagnostic centres across southern Europe and Israel.

**Methods:**

We performed a known sample “ring” trial to compare *L. infantum* quantitative serological tests. Two hundred seventy-two (*n* = 272) canine serum samples of known serological status were chosen from these sites, representative of the region. In-house or commercial ELISA and IFAT were performed according to each laboratory’s specifications. Latent Class Analysis (LCA) was used to determine sensitivity and specificity of each test. True and false positives were calculated to determine the probability of identifying samples.

**Results:**

Sensitivity and specificity for ELISA ranged from 95 to 99% and 92% to 97%, respectively, with moderate variability from one site. Sensitivity and specificity for IFAT ranged from 89 to 99% and 83% to 94%, respectively, with increased variability compared to ELISA. Overall test agreement was 78% with a pair-wise agreement between 65 and 89%.

**Conclusions:**

All sites demonstrated substantial comparative diagnostic accuracy, with good agreement based on known seropositive and seronegative samples. Studies and interventional trials that use these tests will remain valid because of high diagnostic agreement between sites.

**Graphical Abstract:**

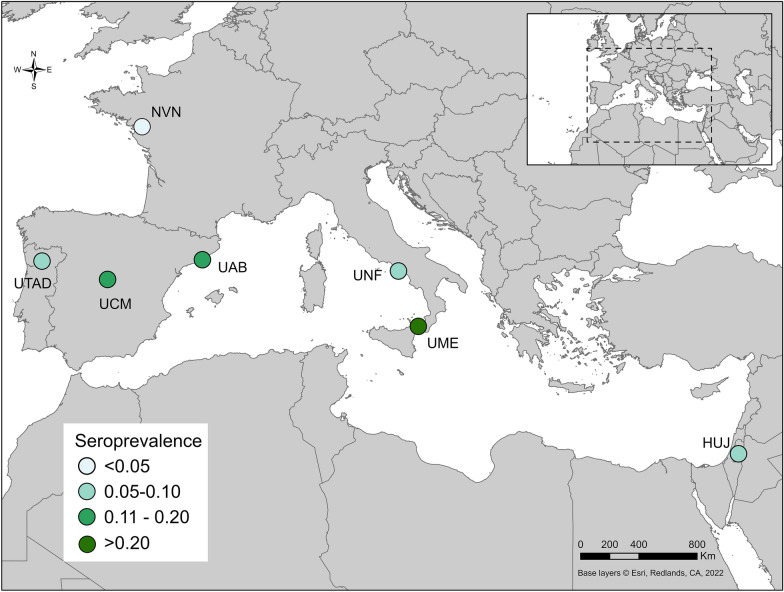

## Background

*Leishmania infantum* is the causative agent for canine leishmaniosis (CanL), a potentially fatal zoonotic vector-borne disease in Southern Europe, Africa, Asia and the Americas [1]. The local environment impacts the ecology of disease, but predominantly dogs are a peridomestic reservoir and phlebotomine sand flies the biological vector [1]. Vertical or transplacental transmission and transmission via blood transfusion also occur [2–4]. Evidence has shown that humans in peri-urban environments with high population density can also serve as a reservoir for *L. infantum* [5]*.*

Seropositivity to *L. infantum* can vary substantially in endemic areas, and not all infected dogs develop disease [6–11]. There is also a spectrum of clinical disease, which can be mild to severe [12]. These presentations, particularly associated with antibody-based pathology or high parasite load, understandably impact detection of *L. infantum* infection. The most frequent clinical signs include skin lesions (78.4% of cases), lymphadenomegaly (64.7% of cases) and weight loss (47.1% of cases) [8]. Clinicopathological abnormalities include mild to moderate non-regenerative anaemia, lymphopenia, hyperproteinaemia, dysproteinaemia and proteinuria. However, these abnormalities occur at varying frequencies, further complicating disease detection [8]. Validating serological diagnostic methods across endemic regions will help establish consistent and reliable *Leishmania* diagnosis for individuals and surveillance across populations.

Seroprevalence of *L. infantum* differs because of variation in sand fly density as well as ecological and environmental variables across endemic areas of Europe and the Mediterranean basin. For example, in Apulia, southern Italy, typically hot and dry, the prevalence of canine *L. infantum* infection was 14.5%, whereas the prevalence in more temperate Tuscany in central Italy was 24% [1]. Seroprevalence in the Alpes Maritimes of France ranged from 3 to 17% dependent on the altitude, humidity, temperature and rainfall in the region [1]. Serological data suggest, of 15 million dogs estimated to live in the Mediterranean basin, roughly 16.7% (2.5 million) were infected with *L. infantum*, and only 10% of those presented with clinical CanL [1, 13]. Different stages of canine infection have been shown to have different levels of infectiousness to sand flies [14, 15]. Effective detection of mild or moderate disease could be of heightened importance in prevention and control of canine leishmaniosis.

Detection of antibodies against *L. infantum* is performed for multiple reasons: to confirm clinical disease, to confirm infection (for blood donors, for breeding or prior to vaccination), to investigate the presence of subclinical infection, to evaluate public health interventions to determine the prevalence of CanL as a sentinel of risk to people and to evaluate the immune response to vaccination in experimental settings [16]. A clinical workup is needed to confirm or exclude clinical leishmaniosis as a diagnosis including routine clinicopathological methods such as complete blood count (CBC), serum biochemical profile and urinalysis [16]. Definitive diagnosis of CanL can be made using microscopic observation of *Leishmania* amastigotes in cytological smears from infected organs and tissues, specifically, bone marrow, lymph nodes, skin or peripheral blood among others [17]. Some of the methods used to obtain samples are overly invasive and generally not ideal for parasite detection from infected healthy dogs [17]. Histopathological analysis of infected organ sections stained by hematoxylin and eosin have also been used to detect parasites and lesions patterns [17]. This method can be very time consuming, and parasitic organisms are difficult to identify and are sometimes non-detectable, resulting in low sensitivity overall [17]. Therefore, *Leishmania* immunohistochemistry with or without quantitative polymerase chain reaction (PCR) is employed in histological samples to increase sensitivity [18]. In vitro culture methods of different tissues can improve the sensitivity of parasitic detection; however, different tissue and organ samples have different parasitic loads, and this can make obtaining sensitive accurate results difficult [17]. Furthermore, this method is not regularly used for diagnosis or monitoring.

Detection of *L. infantum*-specific serum antibodies using quantitative serological techniques, such as the immunofluorescence antibody test (IFAT) or the enzyme-linked immunosorbent assay (ELISA), is essential in diagnosing CanL. IFAT was considered a “gold standard” of serologic diagnosis; however, its application requires a high level of skill and experience, is time consuming and requires specialized laboratory equipment [17]. ELISA is ideal for surveillance because of the ability to screen a large number of samples in a short period of time [17]. High antibody levels have been associated with a higher level of parasitism and presence of more severe disease [19]. Use of quantitative serological techniques that provide an endpoint titre (IFAT) or optical density (OD) reading (ELISA) is recommended to diagnose CanL accurately [20]. The advent of vaccines against *L. infantum* in Europe has raised the possibility of detecting vaccination instead of infection when using serological tests that do not differentiate infected from vaccinated animals (DIVA). This is likely to happen particularly when vaccines contain whole *Leishmania* antigen preparations as immunogens [16].

Given the limitations of microscopy and culture, their use as a gold standard for diagnostic test validation methods is limited. In the absence of a serological gold standard, Latent Class Analysis (LCA) has been used to evaluate diagnostic tests by generating a statistical reference standard [21]. The reference standard is calculated by combining all observed results of the diagnostic tests being evaluated into one large dataset [22]. LCA then uses two different methods, both looking for random effects of why the data might be sorted into classes and performing a handicapped or “penalized” estimation that these classes arose randomly to finalize the results into “classes” or disease states from the data. This allows estimates of sensitivity and specificity to be made from these “true” diagnostic results based on categorization into the most statistically likely classes (positive and negative) [23, 24].

Multi-centred ring trials, or External Quality Control (EQC) sample-based comparison of diagnostic laboratories where a pre-defined set of seropositive and seronegative samples are distributed for testing by each laboratory, have been used to evaluate the comparative performance of diagnostic tests [25, 26]. Ring trial design ensures equal distribution of seropositive and seronegative samples across each site [25, 27]. Few studies have been performed to evaluate the comparative diagnostic accuracy of serological diagnostic tests in Europe, particularly for CanL [28]. Despite efforts by groups such as the LeishVet to develop a consensus of recommendations for the surveillance, prevention and control of CanL [29], comparative validation of *L. infantum* serological tests has not been conducted. Therefore, using LCA, this study aims to compare the diagnostic performance of ELISA and IFAT used by seven veterinary diagnostic laboratories affiliated with LeishVet members (www.leishvet.org) across *L. infantum*-endemic areas of Europe and the Mediterranean basin.

## Methods

**Fig. 1:**
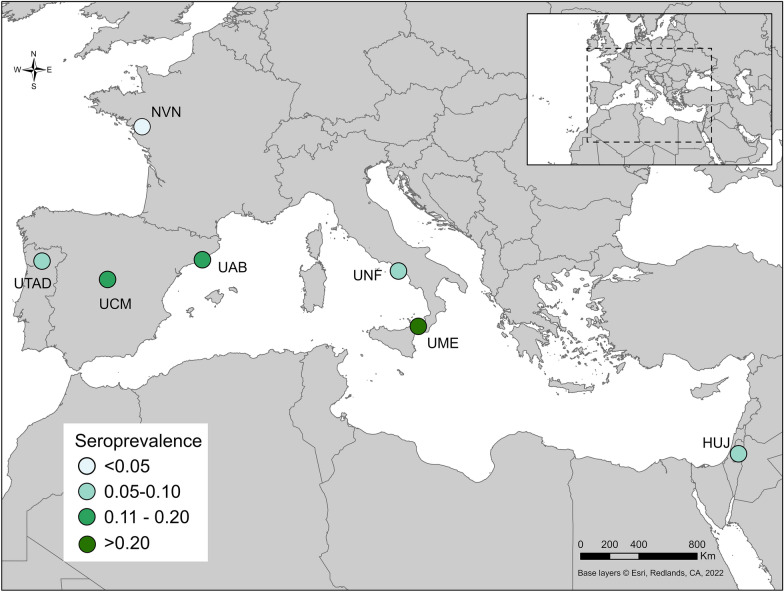
Map of *Leishmania infantum*-endemic areas, location and estimated seroprevalence of all included veterinary diagnostic laboratories. *HUJ* The Hebrew University of Jerusalem, Israel, *NVN* University of Nantes, France, *UAB* Universitat Autònoma de Barcelona, Spain, *UCM* Universidad Complutense de Madrid, Spain, *UME* University of Messina, Italy, *UNF* University of Naples "Federico II", Italy, *UTAD* University of Trás-os-Montes e Alto Douro, Vila Real, Portugal. Content is the intellectual property of Esri and is used herein with permission. Copyright © 2023 Esri and its licensors. All rights reserved

This study performed a diagnostic EQC test to determine the comparative effectiveness of ELISA and IFAT used by seven veterinary diagnostic laboratories (Fig. 1). A total of 272 canine serum samples were analyzed and included in this study. We provided the estimated seroprevalence detected at each location (Fig. 1).

### Sample selection and preparation

Two hundred seventy-two (*n* = 272) canine serum samples were collected, roughly 30 samples from each laboratory’s respective biobank by the LeishVet members in Europe, Israel and the USA (Table 1). These laboratories include Universidad Complutense de Madrid (UCM), École Nationale Vétérinaire de Nantes (NVN), University of Trás-os-Montes e Alto Douro (UTAD), Universitat Autònoma de Barcelona (UAB), University of Naples “Federico II” (UNF), University of Messina (UME) and The Hebrew University of Jerusalem (HUJ) (Table 1).

**Table 1:** Participating locations and diagnostic tests used by each location

LeishVet member—location (code)	Diagnostic test	Cut-off value for a positive sample
Guadalupe Miró—Universidad Complutense de Madrid (UCM), Spain	IFAT	1:100 titre
Christine Petersen—University of Iowa (UOI), USA	NA	NA
Patrick Bourdeau—École National Vétérinaire de Nantes (NVN), France	ELISA, IFAT	> 0.4 OD, 1:80 titre
Luis Cardoso—University of Trás-os-Montes e Alto Douro (UTAD), Portugal	ELISA	> 0.9 units
Laia Solano-Gallego—Universitat Autònoma de Barcelona (UAB), Spain	ELISA	> 28 ELISA units
Gaetano Oliva—University of Naples “Federico II” (UNF), Italy	IFAT	1:160 titre
Maria Grazia Pennisi—University of Messina (UME), Italy	IFAT	1:160 titre
Gad Baneth—The Hebrew University of Jerusalem (HUJ), Israel	ELISA	> 0.2 OD

*ELISA* enzyme-linked immunosorbent assay, *IFAT* immunofluorescence antibody test, *NA* not available, *OD* optical density

Samples were defined as eligible if they had > 1 ml in reserve after other laboratory testing. UCM, NVN, UTAD, UAB, UNF, UME and HUJ provided 30 serum samples; each laboratory provided 23 seropositive samples and 7 seronegative canine serum samples. The University of Iowa (UOI) provided 30 seropositive and 32 seronegative canine samples. Samples collected from UOI represent positive samples from a well-studied population of hunting dogs with *L. infantum* infection, positive on soluble *Leishmania* antigen (SLA) ELISA and by quantitative PCR, established to be autochthonous from vertically infected dogs [2].

Serum samples were collected from each laboratory’s bank of known samples and de-identified for canine patient and providing laboratory at UCM. This process ensured blinded, randomized identification. Each serum sample was divided into seven aliquots, one sample for each participating study location. Prior to being aliquoted, all samples were evaluated for sample quality, to have limited if any haemolysis, at UCM. All samples were either hand carried on dry ice and provided to UCM personnel during a LeishVet meeting or shipped by an overnight carrier within the EU and to Israel. All samples arrived at each location still cold and were immediately stored at – 30 °C.

### Serological test protocol

Each location performed IFAT and/or ELISA specific to its respective site's standard protocol (Table 1). Each laboratory utilized their in-house or commercially generated cut-off values to determine whether a serum sample was positive or negative.

### Sample size

To detect 80% sensitivity and 80% specificity at each study site with an estimated true prevalence at 15% and a marginal error of 0.10, a total of 31 known seropositive canine samples and 31 seronegative canine samples would be needed to achieve 80% power and a 95% significance level to observe a difference. The 32 seronegative samples provided by UOI were collected from a non-endemic region and thus could be used as a true seronegative control group, whereas samples collected from the other study sites could not be confirmed as true-negative samples.

### Outcome variables

Once all serological assays were completed, UOI obtained serological assay results from each study site. Canine serum sample results were dichotomized as positive or negative, dependent on the cut-off value from the test location. Diagnostic positivity or negativity was not altered by UOI researchers after received from collaborators.

### Statistical methods

Two analyses were completed to determine diagnostic test performance and agreement. Cohen's kappa coefficient (κ) was used to compare the levels of agreement between ELISA and IFAT measuring interrater reliability between diagnostic tests. LCA was used to estimate sensitivity, specificity and numbers of true and false positives [30, 31]. κ was performed in R version 3.5.2 and RStudio version 1.3.1093 by comparing the relative observed agreement among test locations, and the hypothetical probability of agreement was calculated using the probabilities of each diagnostic test identifying whether a sample was positive or negative randomly [32]. Observed and estimated levels of agreement were calculated to determine the agreement between each LeishVet group members’ diagnostic results. A contingency table was constructed to calculate agreement between ELISA or IFAT in R version 3.5.2 and RStudio version 1.3.1093. The level of agreement was measured based on the range: κ ≤ 0 indicates no agreement, κ = 0.01–0.20 none to slight, κ = 0.21–0.40 fair, κ = 0.41–0.60 moderate, κ = 0.61–0.80 substantial and κ = 0.81–1.00 almost perfect agreement.

LCA models were run with bootstrapping; expectation-maximization algorithms (EMA) were then used to estimate each test's sensitivity, specificity and true- and false-positive numbers. Ninety-five per cent confidence intervals (CI) were calculated using the sample distribution obtained via bootstrapping [30]. We assumed our data to have two cluster/classes and restricted our model to two classes. Using a random effects model and penalized likelihood estimates, our model generated the marginal probability of a sample based of repeated sampling from the seropositive and seronegative diagnostic results. Samples with the highest marginal probability were classified as the disease samples whereas samples with the lowest marginal probability were defined as non-diseased. After calculating the diseased and non-diseased samples, sensitivity and specificity were established for each diagnostic test at each site. A receiver-operating characteristic (ROC) curve was not performed. We validated the two-class assumption by comparing fit between models assuming two versus three latent classes; we evaluated Alkaike information criterion (AIC) and Bayesian information criterion (BIC) to identify the model with the lowest AIC/BIC and therefore best fit. Latent Class Analysis was conducted in R version 3.5.2 and RStudio version 1.3.1093 using the randomLCA package [30].

Outliers in the data were evaluated utilizing two methods. Initially, sensitivity analysis was performed by rerunning the LCA after excluding irregular values from each location and excluding discordant sites. Once the non-outliner models were in complete agreement, sensitivity, specificity and the true-positive and false-positive numbers were recalculated and compared to the original data that included outliers. AIC and BIC were compared between the model with outlier data and the model without outlier data. Marginal probabilities were examined for the model with outlier data and the model without outlier data to evaluate the extent to which outlier values affected the average probability and thus the model's ability to determine positive and negative samples. Given the sensitivity, specificity and true-positive and false-positive numbers were not different between models, the without outlier model was selected based on higher marginal likelihood estimates. Seroprevalence at each site was mapped in ArcGIS Pro (Esri, Redlands, CA, USA).

### ELISA laboratory methods

NVN, UTAD, UAB and HUJ each performed an ELISA, either an in-house or a commercial ELISA (NVN and UTAD). ELISA was performed in accordance with each laboratory’s specifications [9, 13, 33]. UTAD ELISA was performed in accordance with the Leiscan Leishmania ELISA Test protocols [33]. NVN ELISA was conducted in accordance with the MegaELISA LEISH (MEGACOR Veterinary Diagnostics, Hörbranz, Austria) test protocols [34]. In summary, *Leishmania* antigen was prepared using total *L. infantum* antigen obtained from 3 × freeze-thawed stationary phase *L. infantum* promastigotes (strains utilized are unique to each laboratory) [9, 13]. Microtitre plates were coated with *L. infantum* antigen; antigen source and concentration varied by laboratory and in-house protocol [9, 13]. Plates were incubated overnight at between 2 and 8 ℃ dependent on the laboratory protocol [9, 13]. Canine serum was tested at different dilutions: 1:20 for UTAD, 1:800 for UAB, 1:100 for NVN and 1:1000 for HUJ. For HUJ and UAB protocols, plates were resolved via protein A conjugated to horseradish peroxidase for 1 h at 37 ℃ [9, 13]. Each plate was read when the positive canine reference serum's absorbance (405 nm or 492 nm) reached a pre-determined cut-off value [9, 13]. Results were then determined based on each laboratory’s cut-off value: 0.4 for NVN, 0.8 for UTAD, 0.28 for UAB and 0.2 for HUJ [9, 13, 33].

### IFAT laboratory methods

UCM, NVN, UNF and UME performed in-house IFAT, as such sample preparation and cut-off values and promastigote samples were prepared in accordance with each laboratory [35–38]. In summary, antigens were prepared from *L. infantum* promastigotes (strain MHOM/IT/80/IV74), with preparation conducted by each laboratory in house [35–38]. Canine serum samples were diluted in PBS and added to multisort slides with *L. infantum* antigen and incubated for 30 min at 37 ℃ [35–38]. Fluorescent rabbit anti-dog immunoglobulin G (IgG) antibodies diluted in PBS were added to slides [35–38]. Two-fold serial dilutions specific to each laboratory were performed. Cut-off values were established based on in-house criteria [35–38]. Positive samples are determined based on parasite fluorescence visibility at a cut-off dilution: 1:100 for UCM, 1:80 for NVN and 1:160 for both UNF and UME [35–38].

## Results

**Fig. 2:**
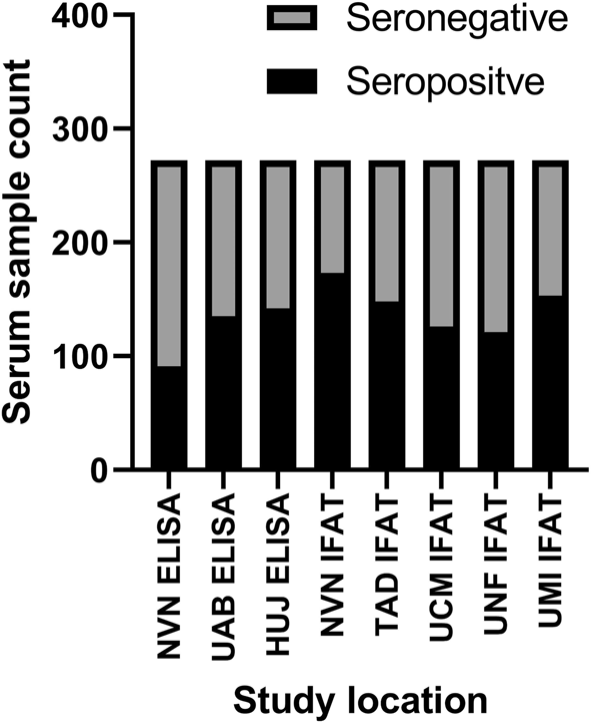
Seropositive samples at each location. Seropositive (black) and seronegative samples (grey) identified by each veterinary diagnostic laboratory. An average of 139 seropositive and 133 seronegative samples were identified. UTAD-ELISA identified the most seropositive samples, 6.5% greater than the average number of seropositive. UNF-IFAT identified the least number of seropositive samples, 13% lower than the average. *ELISA* enzyme-linked immunosorbent assay, *HUJ* The Hebrew University of Jerusalem, Israel, *IFAT* immunofluorescence antibody test, *NVN* University of Nantes, France, *UAB* Universitat Autònoma de Barcelona, Spain, *UCM* Universidad Complutense de Madrid, Spain, *UME* University of Messina, Italy, *UNF* University of Naples "Federico II", Italy, *UTAD* University of Trás-os-Montes e Alto Douro, Vila Real, Portugal

Across 272 samples submitted, 33% of samples (90/272) were positive on all tests, and 18% (48/272) were negative on all tests. Forty-nine per cent (49%) of samples could be considered mixed “seropositive”, i.e. positive on at least one test but not positive by all tests. Sensitivity and specificity values were compared by each test type across the seven laboratories (Fig. 2).

UNF IFAT identified the lowest number of seropositive samples (*n* = 121), 18 samples (13%) fewer than the average number of seropositive samples identified (*n* ~ 139 samples). UTAD identified the highest number of seropositive samples (149 samples), 6.5% greater than the average number of seropositive samples. The four laboratories that utilized ELISA all had very similar sensitivity and specificity (Table 2). NVN, UAB, UTAD and HUJ ELISA all had > 90% sensitivity and specificity, with sensitivity ranging from 95 to 99% and specificity ranging from 92 to 97%. IFAT diagnostic methods also had similar sensitivity (89% to 99%) and specificity (83% to 94%) ranges (Table 3).

**Table 2:** Sensitivity, specificity and 95% confidence interval (CI) for ELISA

Study location code	Sensitivity	95% CI	Specificity	95% CI
NVN	0.99	0.97–1.0	0.95	0.91–0.99
UTAD	0.98	0.96–1.0	0.92	0.86–0.96
UAB	0.95	0.91–0.98	0.97	0.94–1.0
HUJ	0.97	0.94–1.0	0.95	0.91–0.98

*ELISA* enzyme-linked immunosorbent assay, *HUJ* The Hebrew University of Jerusalem, Israel, *NVN* École National Vétérinaire de Nantes (NVN), France, *UAB* Universitat Autònoma de Barcelona, Spain, *UTAD* University of Trás-os-Montes e Alto Douro, Vila Real, Portugal

**Table 3:** Sensitivity, specificity and 95% confidence interval (CI) for IFAT

Study location code	Sensitivity	95% CI	Specificity	95% CI
NVN	0.98	0.94–1.0	0.93	0.89–0.98
UCM	0.89	0.83–0.94	0.94	0.90–0.98
UNF	0.94	0.89–0.98	0.88	0.82–0.94
UME	0.99	0.97–1.0	0.83	0.76–0.90

*IFAT* immunofluorescence antibody test, *NVN* École National Vétérinaire de Nantes (NVN), France, *UCM* Universidad Complutense de Madrid, Spain, *UNF* University of Naples "Federico II", Italy, *UME* University of Messina, Italy

**Fig. 3:**
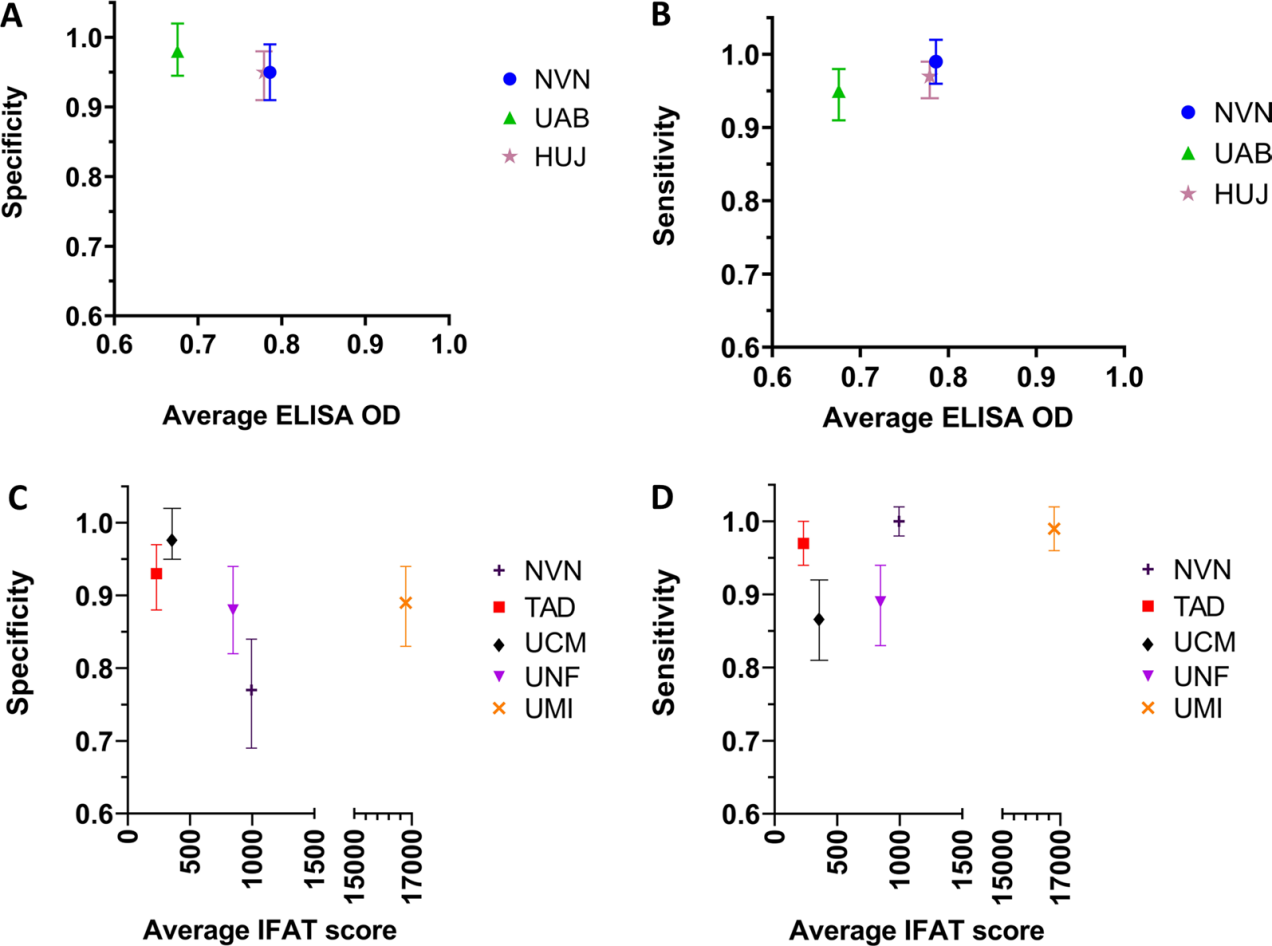
Sensitivity and specificity range for veterinary diagnostic laboratories generated by Latent Class Analysis. **A** Specificity range of ELISA used by average ELISA OD/units. **B** Sensitivity range of all ELISA used by average ELISA OD/units. **C** Specificity range of the IFAT used by average IFAT titre value. **D** Sensitivity range of the IFAT used by average IFAT titre value. Sensitivity was calculated as the number true-positive samples divided by the number of true-positive and false-negative samples for each diagnostic. Specificity was calculated as the number of true-negative samples divided by the number true-negative and false-positive samples. *ELISA* enzyme-linked immunosorbent assay, *HUJ* The Hebrew University of Jerusalem, Israel, *IFAT* immunofluorescence antibody test, *NVN* University of Nantes, France, *OD* optical density, *UAB* Universitat Autònoma de Barcelona, Spain, *UCM* Universidad Complutense of Madrid, Spain, *UME* University of Messina, Italy, *UNF* University of Naples "Federico II", Italy, *UTAD* University of Trás-os-Montes e Alto Douro, Vila Real, Portugal

LCA was used to compare the sensitivity and specificity from each study site to an estimated gold standard (Fig. 3). NVN and HUJ had substantial overlap in average ELISA OD and their specificity (Fig. 3A), whereas UAB had lower average ELISA units but still with considerable overlap with NVN and HUJ. UTAD had the highest average ELISA OD, but specificity still overlapped with another ELISA. NVN and HUJ overlapped in both average ELISA OD and sensitivity, and UAB had the lower average ELISA units, but its sensitivity range overlapped with NVN and HUJ (Fig. 3B). UTAD had the highest average ELISA OD, but its sensitivity range still overlapped with all the other ELISAs. The specificity range for all four IFAT diagnostics all overlapped substantially, with NVN and UCM possessing the greatest overlap (Fig. 3C). The average IFAT scores did not overlap for any of the four IFAT diagnostic laboratories. The sensitivity range for all four IFATs overlapped, but NVN and UME had the greatest level of overlap (Fig. 3D). While the specificity range for each diagnostic laboratory showed some overlap, they were not as closely clustered compared to ELISA OD or units. The average IFAT titre score ranged from < 500 to as high as 17,000. The distribution of IFAT titres showed that three of the four diagnostic laboratory IFATs ranged from 0 to > 1:5120.

**Fig. 4:**
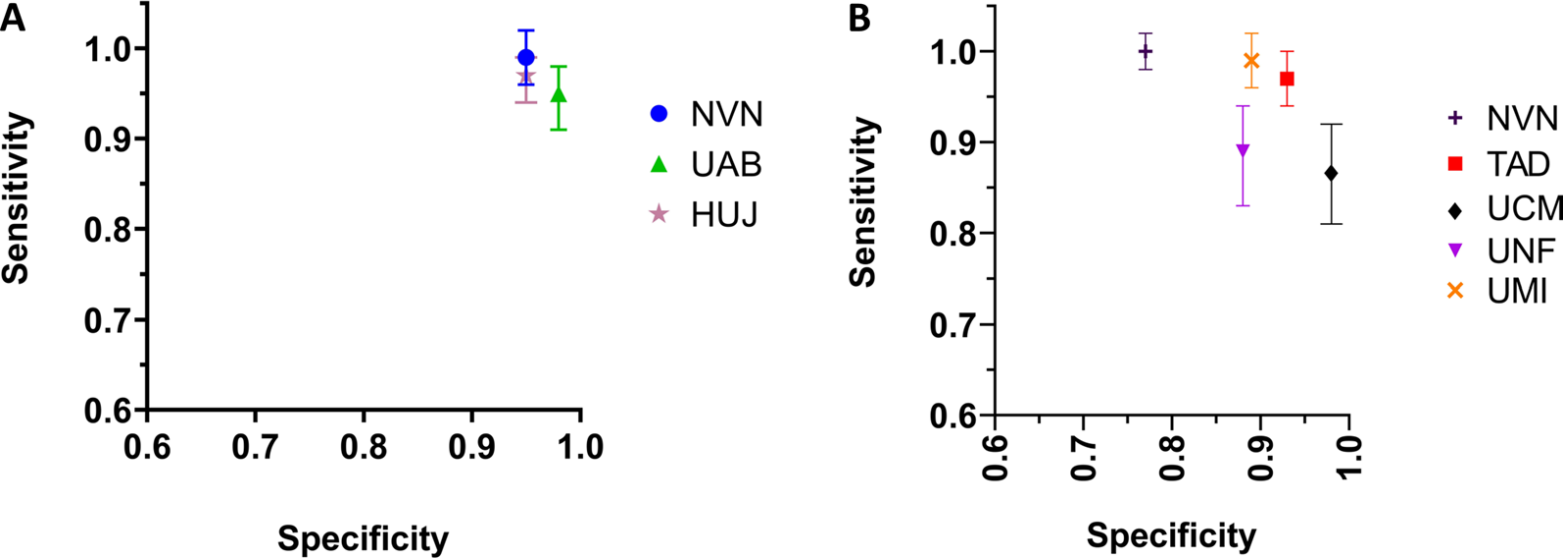
Sensitivity by specificity plot for ELISA and IFAT. **A** ELISA sensitivity by specificity. **B** IFAT sensitivity by specificity. Sensitivity was calculated as the number true-positive samples divided by the number of true-positive and false-negative samples for each diagnostic. Specificity was calculated as the number of true-negative samples divided by the number true-negative and false-positive samples. *ELISA* enzyme-linked immunosorbent assay, *HUJ* The Hebrew University of Jerusalem, Israel, *IFAT* immunofluorescence antibody test, *NVN* University of Nantes, France, *UAB* Universitat Autònoma de Barcelona, Spain, *UCM* Universidad Complutense of Madrid, Spain, *UME* University of Messina, Italy, *UNF* University of Naples "Federico II", Italy, *UTAD* University of Trás-os-Montes e Alto Douro, Vila Real, Portugal

Comparing sensitivity to specificity between ELISA and IFAT, there was a similar trend in overlap among ELISA results (Fig. 4A) and dispersion across IFAT results (Fig. 4B). Comparing ELISA sensitivity with specificity, all four tests results clustered between 90 and 100% sensitivity and specificity. Notably, we see that NVN and HUJ grouped closely together in both sensitivity and specificity, while UAB and UTAD grouped closer together (Fig. 4A). Overall, there was good sensitivity and specificity for all four ELISA diagnostics. Less grouping of IFAT was seen in sensitivity and specificity. Sensitivity in UME and NVN IFAT ranged from 90 to 100%, whereas UNF and UCM sensitivity ranged from 80 to 95%. Specificity ranged from 75 to 100%. The sensitivity and specificity range of IFAT was notably larger than sensitivity and specificity range of ELISA.

**Fig. 5:**
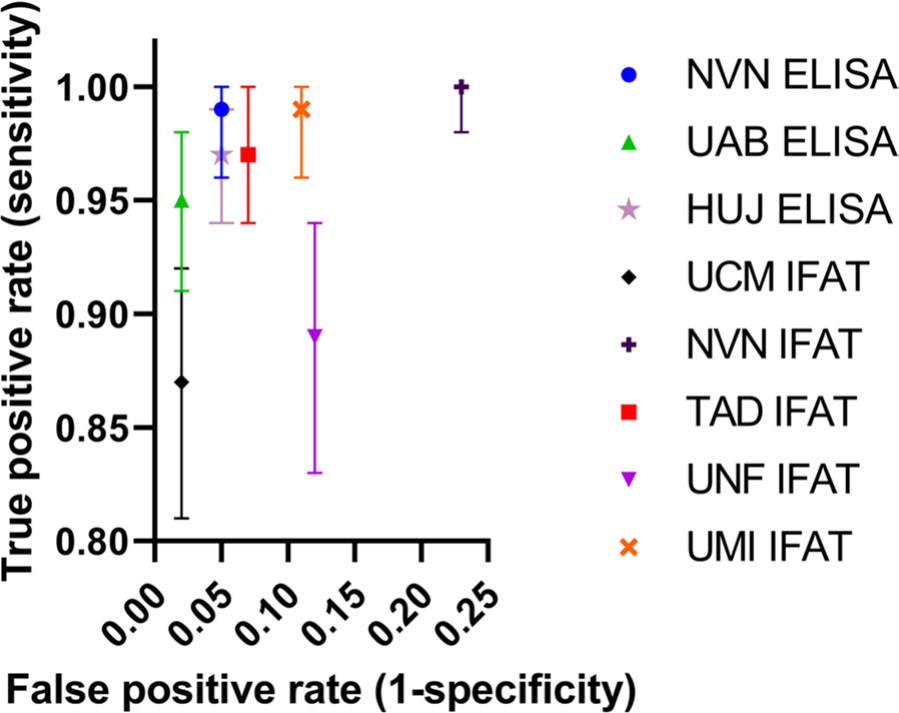
True positives by false positives for all seven veterinary diagnostic laboratories. True-positive percentage (rate) was based of the sensitivity, and the false-positive percentage (rate) was calculated as one minus specificity. *ELISA* enzyme-linked immunosorbent assay, *HUJ* The Hebrew University of Jerusalem, Israel, *IFAT* immunofluorescence antibody test, *NVN* University of Nantes, France, *UAB* Universitat Autònoma de Barcelona, Spain, *UCM* Universidad Complutense of Madrid, Spain, *UME* University of Messina, Italy, *UNF* University of Naples "Federico II", Italy, *UTAD* University of Trás-os-Montes e Alto Douro, Vila Real, Portugal

NVN IFAT had the highest true-positive proportion (~ 100%) and thus the best ability to detect seropositive canine samples (Fig. 5). UCM IFAT had the lowest true-positive proportion (87%), while UME IFAT had the highest proportion of false positives at 17%. UAB ELISA had the lowest proportion of false positives at 3%, suggesting that UAB was least likely to identify a negative canine sample as positive (Fig. 5). Overall, all the diagnostic laboratories had a relatively low proportion of false positives at < 20% and a proportion of true positives > 80% (Fig. 5). All seven diagnostic laboratories showed substantial overall agreement (κ = 0.78, where κ = 0.61–0.80 is substantial agreement) when comparing ELISA and IFAT (Table 4), with an overall agreement of 78%. IFAT had constantly lower levels of agreement when compared to ELISA.

**Table 4:** Cohen’s kappa coefficient (κ) for the individual serological tests

NVN − ELISA	—							
UTAD − ELISA	0.87	—						
UAB − ELISA	0.88	0.82	—					
HUJ − ELISA	0.87	0.87	0.87	—				
NVN − IFAT	0.83	0.79	0.85	0.85	—			
UCM − IFAT	0.78	0.75	0.84	0.75	0.74	—		
UNF − IFAT	0.74	0.69	0.75	0.73	0.78	0.67	—	
UME − IFAT	0.80	0.78	0.77	0.78	0.75	0.73	0.65	—
	NVN − ELISA	UTAD − ELISA	UAB − ELISA	HUJ − ELISA	NVN − IFAT	UCM − IFAT	UNF − IFAT	UME − IFAT

The level of agreement based on κ was interpreted according to the following assumptions: < 0.2 = slight; 0.2–0.4 = fair; 0.4–0.6 = moderate; 0.6–0.8 = substantial; > 0.8 = almost perfect agreement

*ELISA* enzyme-linked immunosorbent assay, *HUJ* The Hebrew University of Jerusalem, Israel, *IFAT* immunofluorescence antibody test, *NVN* École National Vétérinaire de Nantes, France, *UAB* Universitat Autònoma de Barcelona, Spain, *UCM* Universidad Complutense de Madrid, Spain, *UME* University of Messina, Italy, *UNF* University of Naples "Federico II", Italy, *UTAD* University of Trás-os-Montes e Alto Douro, Vila Real, Portugal

## Discussion

This study evaluated the diagnostic sensitivity, specificity and true-positive and false-positive numbers and the agreement of seven southern European and Israeli veterinary diagnostic tests for antibodies against *L. infantum* antigen in blinded, shared, canine serum samples. Using LCA, we established an estimated serological gold standard for which to compare ELISA and IFAT. Our study found that ELISA had good sensitivity specificity and an overall high number of true positives. IFAT also had a high number of true positives but less agreement. Notably, the ELISA sensitivity ranged from 95 to 99%, while the specificity ranged from 92 to 97%. These results were greater than those presented by Solcà et al. [39] whose in-house ELISA had a sensitivity of 79.2% (95% CI 68.0−90.3%) and a specificity of 90.6% (95% CI 88.6–92.6%). Machado de Assis et al. [40] reported a similar sensitivity range of 96.3–99.6%, but a slightly lower specificity range of 75.0–88.3% for rK39-ELISA compared to ELISA conducted in our study. The ELISA diagnostic laboratories in the present study were similarly accurate at identifying canine seropositive and seronegative samples for *L. infantum*.

IFAT sensitivity ranged from 89 to 99%, while the specificity ranged from 83 to 94%, similar to sensitivity (range: 84.0–92.0%) and specificity (range: 75.0–88.2%) of the commercial kit used by Machado de Assis et al. [40]. IFATs performed by laboratories in Europe and the Mediterranean basin were similarly effective at identifying seropositive and seronegative canine samples. Overall agreement for ELISA and IFAT was 78%, comparable to the overall agreement calculated by Basurco et al. [34] at 80% agreement. Notably, Basurco et al. [34] only used one in-house diagnostic test, whereas the tests assessed in this study were predominantly in-house assays with two commercial kits. This suggests that in-house serological tests generated comparable values to validated commercial kit tests [34]. Therefore, not only are sensitivity and specificity results presented herein like those seen in other studies that used LCA, but so were the overall agreement levels [34, 39, 40].

When comparing the closeness of measurements to the LCA estimated serological gold standard, defined as accuracy, and the closeness of the measurement to each other, i.e. precision, it is essential to consider the true- and false-positive numbers for each test. IFAT was more likely to identify seropositive canine samples as seronegative overall compared to ELISA. Furthermore, we can determine how likely each diagnostic test is to detect a false-negative sample incorrectly from this comparison (Fig. 5). IFAT had a higher overall false-positive number than ELISA, likely due to reporter bias from human error in subjectively “calling” a sample positive at a specific dilution and methodological differences in how this occurs. Beyond reporter observation error-based differences, there are many other diagnostic method-based potential reasons for differences in likelihood to call a sample positive for both IFAT and ELISA. IFAT analytically sacrificed the ability to identify seronegative samples to detect all seropositive samples in this study. In contrast, ELISA had a lower false-positive number overall; the lowest and highest false-positive number only differed by 5%. This is likely because ELISA is based on machine measurements to identify positive samples removing human bias to a greater extent than IFAT.

While both diagnostic tests are relatively accurate, ELISA had greater accuracy overall due to the higher level of agreement. Discordance in accuracy between ELISA and IFAT is due to the methodology of ELISA versus IFAT [34, 40]. There is not a reference standard across countries where CanL is endemic, and positive control samples obtained in Italy could present different fluorescence titres compared to control samples obtained in France or Spain, for example [34]. A similar effect was observed with the average IFAT titre score between each veterinary diagnostic laboratory. ELISA may also have greater standardization compared to the IFAT and often have similar cut-off values from country to country. Indeed, we see this in our results as the diagnostic laboratory results cluster tighter around average ELISA OD units.

Although our study effectively compared seven veterinary diagnostic laboratories, there were limitations to this study. Specifically, a multinational study with laboratories from multiple regions increases the generalizability of our results. However, each laboratory uses slightly different diagnostic methods, thus introducing observation bias. For example, an IFAT diagnostic result was determined to be seronegative or seropositive dependent on a laboratorian’s observations. Therefore, this method introduced observational variability between each laboratory's IFAT. The purpose of the study was not to harmonize diagnostic methods across these laboratories as they must follow the regulations of different regulatory agencies for each country (France, Israel, Italy, Portugal and Spain). If this was not the case, we could consider evaluating specific parts of protocols for instance using the same canine antibody clones and lots, considering types of microscopes (IFAT) or plate readers (ELISA), and other method-based means to bring further diagnostic similarity across sites.

Using LCA, our study found that ELISAs used by the veterinary diagnostic laboratories were able to produce very similar results despite disparate locations. IFAT produced similar results with more variability between locations. Overall, ELISA and IFAT for CanL across seven test sites were able to produce similar results regardless of methodology or location, which means the concerned laboratories are validated for the two quantitative "diagnostic” serological methods.

## Conclusions

To our knowledge, this study is the first to objectively evaluate the two reference laboratory techniques for the quantitative serological diagnosis of CanL at the international level in endemic areas of five countries. Their performances are comparable and validated and their respective benefits measured. This work can be useful for developing future clinical or epidemiological work around this platform, proposing an accurate serological diagnosis or carrying out serological monitoring of *L. infantum* infection in dogs from one country to another.

## Data Availability

No datasets were generated or analysed during the current study.

## Abbreviations

AIC: Alkaike information criterion
BIC: Bayesian information criterion
CanL: Canine leishmaniosis
CI: Confidence interval
ELISA: Enzyme-linked immunosorbent assay
EQC: External quality control
HUJ: The Hebrew university of Jerusalem
IFAT: Immunofluorescence antibody test
LCA: Latent class analysis
NVN: École Nationale Veterinaire de Nantes
OD: Optical density
UAB: Universitat autònoma de Barcelona
UCM: Universidad complutense de Madrid
UNF: University of Naples “Federico II”
UME: University of Messina
UTAD: University of Trás-os-Montes e Alto Douro
